# Lung Cancer Screening Prevalence and Changes in 2024

**DOI:** 10.1001/jamainternmed.2026.0493

**Published:** 2026-04-27

**Authors:** Todd Burus, Caree R. McAfee, Jennifer Redmond Knight, Timothy W. Mullett, Pamela C. Hull

**Affiliations:** 1 Markey Cancer Center, University of Kentucky, Lexington; 2 Department of Internal Medicine, College of Medicine, University of Kentucky, Lexington; 3 College of Public Health, University of Kentucky, Lexington; 4 Kentucky Cancer Consortium, Lexington; 5 Division of Cardiothoracic Surgery, College of Medicine, University of Kentucky, Lexington; 6 Department of Behavioral Science, College of Medicine, University of Kentucky, Lexington

## Abstract

This cross-sectional study compares up-to-date lung cancer screening prevalence and changes.

The US Preventive Services Task Force (USPSTF) recommends annual lung cancer screening for certain high-risk individuals to help reduce lung cancer morbidity and mortality in the US.^1^ Over its first decade of use, however, screening uptake has remained low.^2^ In this cross-sectional study, we sought to compare up-to-date (UTD) lung cancer screening prevalence in 2024 across various groups and states and to assess changes since 2022.

## Methods

We analyzed data from the 2024 Behavioral Risk Factor Surveillance System (BRFSS) national dataset—a population-based, state-representative survey of health behaviors—and compared results with those of the 2022 dataset. As a deidentified, public-use dataset, the BRFSS requires no institutional review board approval. We followed the Strengthening the Reporting of Observational Studies in Epidemiology (STROBE) reporting guideline.

We calculated UTD screening prevalence in 2024 (self-report of computed tomography to “scan or check for lung cancer” within the past year) among eligible individuals who (1) were aged 50 to 80 years, (2) were current smokers or have quit within 15 years, and (3) had a smoking history of 20 or more pack-years (eMethods in the Supplement 1). Temporal comparisons were restricted to those aged 50 through 79 years due to a known coding issue from 2022.^2^

Adjusted prevalence ratios (aPRs) with 95% CIs were estimated from logistic regression models for subgroup comparisons in 2024.^3^ Adjusted differences from 2022 were also measured. Models were adjusted for sex, age, self-reported race and ethnicity, education, insurance status, and urbanicity. The sample was restricted to individuals with complete data on smoking, tomographic scan history, and covariates. Analysis was performed in R version 4.5.1 (R Core Team) and incorporated complex sampling weights using the survey package.

## Results

Our sample included 26 104 respondents who met USPSTF eligibility criteria, representing 12.3% of US adults aged 50 to 80 years. Of the eligible respondents, 12 735 (45.6%) were female and 13 369 (54.4%) were male. Nationally, 24.49% (n = 6903) of eligible respondents were UTD on their screenings in 2024 (Table). UTD prevalence increased with age—from 11.32% among those aged 50 through 54 years to 31.20% among those aged 75 through 79 years. Compared with those with private insurance, UTD prevalence was significantly higher among individuals with military-related coverage (aPR, 1.83; 95% CI, 1.52-2.20), Medicare (aPR, 1.28; 95% CI, 1.12-1.45), and Medicaid (aPR, 1.23; 95% CI, 1.04-1.46), and significantly lower among those without insurance (aPR, 0.37; 95% CI, 0.21-0.64).

**Table.** Prevalence of Up-to-Date Lung Cancer Screening Status by Population Subgroups In 2024 and Changes From 2022, Behavioral Risk Factor Surveillance System, 2024

Variable	Lung cancer screening in 2024			Change from 2022, percentage point (95% CI)^c^
	Sample, No. (weighted %)	Up-to-date prevalence, weighted % (95% CI)^a^	Group difference in 2024, adjusted prevalence ratio (95% CI)^b^	
Total eligible, No.^d^	29 789	NA	NA	NA
With complete data, No.^e^	26 104	24.49 (23.37-25.65)	NA	6.00 (4.54-7.44)
Sex				
Female	12 735 (45.6)	24.78 (23.21-26.39)	1.01 (0.93-1.11)	6.57 (4.48-8.66)
Male	13 369 (54.4)	24.26 (22.66-25.91)	1 [Reference]	5.39 (3.42-7.36)
Age, y				
50-54	3236 (16.8)	11.32 (8.81-14.26)	0.47 (0.36-0.61)	4.52 (1.69-7.35)
55-59	4136 (17.7)	19.45 (17.09-21.99)	0.76 (0.63-0.91)	6.44 (3.63-9.25)
60-64	5732 (24.6)	23.99 (21.86-26.22)	0.93 (0.79-1.09)	5.40 (2.44-8.35)
65-69	5798 (19.2)	32.78 (29.85-35.81)	1.09 (0.94-1.26)	9.09 (5.55-12.63)
70-74	4335 (13.3)	32.58 (29.55-35.72)	1.06 (0.90-1.23)	6.77 (3.11-10.43)
75-79	2867 (8.4)	31.20 (27.41-35.19)	1 [Reference]	1.63 (−4.38 to 7.64)
Race and ethnicity^f^				
Non-Hispanic				
American Indian/Alaska native	539 (1.9)	30.80 (20.51-42.72)	1.24 (0.90-1.71)	12.06 (3.01-21.11)
Asian	146 (2.2)	19.78 (7.38-38.76)	0.81 (0.41-1.58)	1.83 (−11.41 to 15.06)
Black	1146 (7.3)	23.98 (19.9-28.46)	0.90 (0.75-1.08)	3.59 (−1.79 to 8.98)
White	22 649 (78.0)	25.03 (23.89-26.19)	1 [Reference]	6.17 (4.68-7.66)
Other race	898 (4.2)	20.66 (15.52-26.60)	0.87 (0.67-1.14)	9.58 (4.05-15.12)
Hispanic	726 (6.5)	20.85 (14.4-28.60)	0.97 (0.72-1.30)	3.50 (−4.69 to 11.68)
Educational attainment				
<High school	2496 (15.2)	20.36 (17.69-23.24)	0.80 (0.68-0.95)	4.62 (1.20-8.04)
High school or GED	9490 (33.9)	25.07 (23.23-26.98)	0.94 (0.83-1.06)	5.86 (3.46-8.27)
Some college	8831 (36.3)	24.69 (22.67-26.79)	0.91 (0.81-1.02)	5.84 (3.28-8.40)
College graduate	5287 (14.6)	26.98 (24.22-29.88)	1 [Reference]	8.56 (5.14-11.98)
Urbanicity				
Metropolitan county	16 479 (77.7)	24.69 (23.34-26.08)	1.07 (0.98-1.17)	5.74 (4.00-7.48)
Nonmetropolitan county	9625 (22.3)	23.80 (21.96-25.71)	1 [Reference]	6.96 (4.70-9.22)
Health insurance type				
Private	7159 (32.4)	18.98 (17.18-20.88)	1 [Reference]	4.64 (2.23-7.05)
Medicare	12 047 (40.1)	30.63 (28.79-32.52)	1.28 (1.12-1.45)	6.52 (4.04-9.00)
Medicaid	2589 (9.9)	23.64 (20.08-27.50)	1.23 (1.04-1.46)	6.76 (2.72-10.80)
Military-related	1633 (6.3)	39.05 (33.54-44.77)	1.83 (1.52-2.20)	15.69 (9.35-22.04)
Other public	1574 (6.2)	15.90 (13.06-19.07)	0.82 (0.67-1.00)	1.46 (−2.62 to 5.54)
Uninsured	1102 (5.3)	6.07 (3.01-10.73)	0.37 (0.21-0.64)	2.35 (−0.29 to 5.00)

Abbreviations: GED, General Educational Development; NA, not applicable.

^a^ Includes 49 states (except Tennessee due to small sample size), the District of Columbia, Puerto Rico, Guam, and the US Virgin Islands.

^b^ Adjusted prevalence ratios with 95% CIs were estimated using logistic regression with average marginal predictions. Models were adjusted for sex, age group, race and ethnicity, educational attainment, insurance status, and urbanicity.

^c^ Restricted to ages 50 through 79 years due to known issue with survey coding in 2022. Models were adjusted for sex, age, self-reported race and ethnicity, educational attainment, insurance status, and urbanicity.

^d^ Eligible individuals were aged 50 to 80 years who currently smoke or formerly smoked (<15 years since quitting), with a 20 or more pack-year smoking history.

^e^ Respondents with missing data on history of smoking or tomographic scans as well as adjustment variables were excluded.

^f^ Information on race and ethnicity was self-reported by participants according to available categories in the survey. Other non-Hispanic races includes individuals of Native Hawaiian or other Pacific Islander, multiple races, or unknown race.

UTD screening prevalence increased 6.00 percentage points since 2022, with significant gains across most subgroups and no declines. UTD prevalence increased by 4.52 percentage points among adults aged 50 through 54 years, who were first made eligible for screening in 2021, and by 15.69 percentage points among individuals with military-related insurance. From 2022, no change in prevalence was observed among uninsured individuals, the oldest eligible age group, or Hispanic ethnicity or non-Hispanic Asian or non-Hispanic Black individuals.

State-level UTD prevalence in 2024 ranged from 13.43% in South Dakota to 38.36% in Massachusetts, with the highest prevalences clustered in the Northeast region (Figure). Twenty-one states had significant increases since 2022, spread evenly across regions. No state had significant declines.

**Figure.**
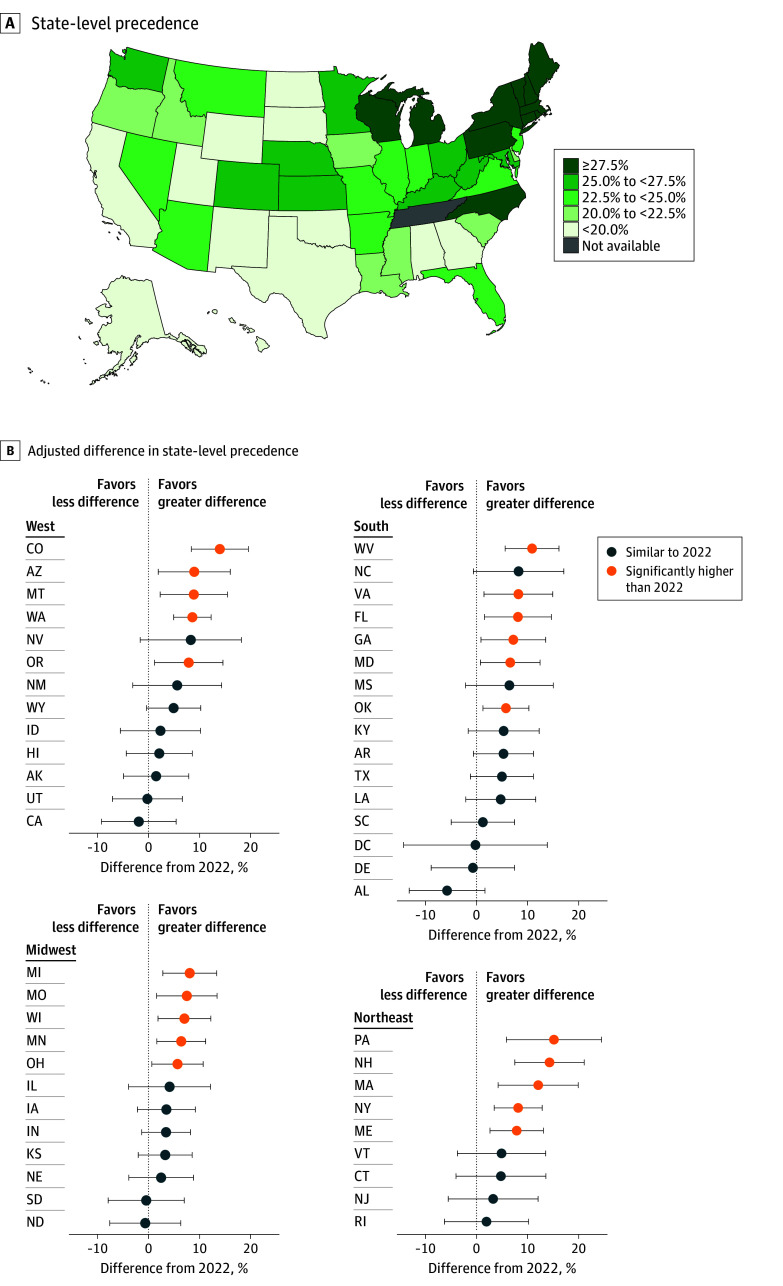
State-Level Prevalence Map of Up-to-Date Lung Cancer Screening Status and Forest Plot of Changes From 2022 A, State-level prevalence map of up-to-date lung cancer screening status among eligible individuals in the Behavioral Risk Factor Surveillance System, 2024. B, Forest plots of adjusted difference in state-level prevalence from 2022. The US Preventive Services Task Force recommends annual screening with low-dose computed tomography for lung cancer for all individuals aged 50 to 80 years who currently smoke or formerly smoked (<15 years since quitting), with a 20 or more pack-year smoking history. Difference from 2022 restricted to ages 50 through 79 years due to a known coding issue in 2022 and adjusted for age, sex, self-reported race and ethnicity; educational attainment; insurance status; and urbanicity. Differences (dots) presented with 95% CIs (whiskers). State-level up-to-date screening status prevalence in 2024 was deemed significantly different from that in 2022 if the 95% CI for the adjusted difference did not contain 0.

## Discussion

In this cross-sectional study, approximately 1 in 4 screening-eligible adults were UTD on lung cancer screening in 2024, with a 6–percentage point increase since 2022. Improvements were uneven, with no change among Hispanic, non-Hispanic Asian, or non-Hispanic Black adults. Culturally tailored outreach through community health workers and patient navigation may help increase screening uptake in these groups. The strong association of UTD status with military-related vs private insurance coverage suggests that investments in comprehensive lung cancer screening programming and strategic partnerships may be effective in driving screening completion.^4^ State-level variation in prevalence and change over time provide a useful context for evaluating the potential impact of laws establishing lung cancer screening programs and other related measures.^5^

Study limitations include biases inherent to self-reported data and exclusion of adults aged 80 years in temporal analysis. Differences in survey design, questions, and mode of administration may lead to discrepancies between national surveys estimating screening status.^6^

Despite improvements, adherence to annual lung cancer screening remained low and uneven in 2024, underscoring the need to strengthen and expand programs and policies that increase uptake among eligible adults.

## Notes

Supplement 1. **eMethods.** Calculating up-to-date (UTD) lung cancer screening (LCS) status in the 2024 Behavioral Risk Factor Surveillance System (BRFSS)

Supplement 2.
Data Sharing Statement

## References

[1] Krist AH, Davidson KW, Mangione CM, ; US Preventive Services Task Force. Screening for lung cancer: US Preventive Services Task Force recommendation statement. JAMA. 2021;325(10):962-970. doi:10.1001/jama.2021.1117 33687470

[2] Bandi P, Star J, Ashad-Bishop K, Kratzer T, Smith R, Jemal A. Lung cancer screening in the US, 2022. JAMA Intern Med. 2024;184(8):882-891. doi:10.1001/jamainternmed.2024.1655 38856988 PMC11165414

[3] Graubard BI, Korn EL. Predictive margins with survey data. Biometrics. 1999;55(2):652-659. doi:10.1111/j.0006-341X.1999.00652.x 11318229

[4] Maurice NM, Tanner NT. Lung cancer screening at the VA: past, present and future. Semin Oncol. 2022;49(3-4):206-212. doi:10.1053/j.seminoncol.2022.06.001 35831214

[5] Piekarz-Porter E, Kim SJ. State law at the intersection of lung cancer screening guidelines and social determinants of health. J Cancer Policy. 2025;43:100561. doi:10.1016/j.jcpo.2025.100561 39922310 PMC13173492

[6] Sauer AG, Liu B, Siegel RL, Jemal A, Fedewa SA. Comparing cancer screening estimates: Behavioral Risk Factor Surveillance System and National Health Interview Survey. Prev Med. 2018;106:94-100. doi:10.1016/j.ypmed.2017.10.019 29079098

