# Supplementary material for: Lung Cancer Screening Prevalence and Changes in 2024
Source: JAMA Intern Med. 2026 Apr 27;186(6):775–8. doi: 10.1001/jamainternmed.2026.0493 (PMC13122494; doi:10.1001/jamainternmed.2026.0493)
Supplement: Supplement 1. — eMethods. Calculating up-to-date (UTD) lung cancer screening (LCS) status in the 2024 Behavioral Risk Factor Surveillance System (BRFSS) [file jamainternmed-e260493-s001.pdf]

Supplemental Online Content

Burus T, McAffe CR, Knight JR, Mullet TW, Hull PC. Estimates of and changes in lung cancer screening prevalence in the United States, 2024. *JAMA Internal Medicine*. Published online April 27, 2026.
doi:10.1001/jamainternmed.2026.0493

eMethods. Calculating up-to-date (UTD) lung cancer screening (LCS) status in the 2024 Behavioral Risk Factor Surveillance System (BRFSS)

This supplementary material has been provided by the authors to give readers additional information about their work.

© 2026 Burus T, et al. *JAMA Internal Medicine*.

eMethods. Calculating up-to-date (UTD) lung cancer screening (LCS) status in the 2024 Behavioral Risk Factor Surveillance System (BRFSS)

Respondents to the 2024 BRFSS were asked several questions to determine their eligibility for LCS and whether they were UTD with US Preventive Services Task Force (USPSTF) recommendations for receipt of annual screening with a low-dose computed tomography (CT) scan. Responses to individual questions were recorded and, in some instances, combined into calculated variables by staff at the Centers for Disease Control and Prevention.

The 2024 BRFSS national dataset includes a calculated variable _LCSPSTF for indicating whether an individual met USPSTF guidelines for LCS. This variables categorizes respondents as (1) meeting USPSTF guidelines, (2) not meeting USPSTF guidelines, or (3) “not eligible, don’t know/not sure, refused, or missing”. This categorization is based on values from two other calculated variables: _LCSELIG, which indicates whether an individual was eligible for LCS, and _LCSCTSN, which indicates when an individual last received a chest CT scan for lung cancer.

According to the BRFSS codebook, individuals were marked as meeting USPSTF guidelines for _LCSPSTF if they were eligible for LCS according to _LCSELIG **and** had a chest CT scan for lung cancer in the previous 12 months. They were marked as not meeting USPSTF guidelines for _LCSPSTF if they were eligible for LCS according to _LCSELIG **but** (1) had their last chest CT scan for lung cancer outside the previous 12 months, or (2) only had a chest CT scan in the previous 12 months for something other than lung cancer, or (3) had never had a chest CT scan for lung cancer. They were marked as “not eligible, don’t know/not sure, refused, or missing” for _LCSPCTF for all other combinations of responses to _LCSELIG and _LCSCTSN.

Unfortunately, there was an error in the _LCSPSTF variable reported in the 2024 BRFSS national dataset at the time of our analysis; namely, individuals marked as eligible for LCS according to _LCSELIG and with a missing value for _LCSCTSN were incorrectly classified as not meeting USPSTF guidelines (instead of as “not eligible, don’t know/unsure, refused, or missing”). This error affected 2,187 respondents and resulted in an approximately 1.5 percentage point reduction in estimated prevalence of UTD LCS screening status. To correct this error, we prepared a new calculated variable indicating whether an individual met USPSTF guidelines for LCS with the appropriate criteria, which we labeled _LCSPSTF80. All estimates for UTD LCS status in 2024 in this study were based on the corrected variable.

© 2026 Burus T, et al. *JAMA Internal Medicine*.
