# Supplementary material for: Lung Cancer Screening Prevalence and Changes in 2024
Source: JAMA Intern Med. 2026 Apr 27;186(6):775–8. doi: 10.1001/jamainternmed.2026.0493 (PMC13122494; doi:10.1001/jamainternmed.2026.0493)
Supplement: Supplement 2. — Data Sharing Statement [file jamainternmed-e260493-s002.pdf]

Data Sharing Statement

Burus. Estimates of and Changes in Lung Cancer Screening Prevalence in the United States, 2024. *JAMA Intern Med*. Published April 27, 2026. doi:10.1001/jamainternmed.2026.0493

Data
Data available: No

Additional Information
Explanation for why data not available: Data is publicly available from the Centers for Disease Control and Prevention.
